# Mapping the distribution of the main host for plague in a complex landscape in Kazakhstan: An object-based approach using SPOT-5 XS, Landsat 7 ETM+, SRTM and multiple Random Forests

**DOI:** 10.1016/j.jag.2012.11.007

**Published:** 2013-08

**Authors:** L.I. Wilschut, E.A. Addink, J.A.P. Heesterbeek, V.M. Dubyanskiy, S.A. Davis, A. Laudisoit, M.Begon, L.A. Burdelov, B.B. Atshabar, S.M. de Jong

**Affiliations:** a Utrecht University, Department of Physical Geography, Heidelberglaan 2, PO Box 80115, 3508 TC Utrecht, The Netherlands; b Utrecht University, Faculty of Veterinary Medicine, Yalelaan 7, 3584 CL Utrecht, The Netherlands; c Stavropol Plague Control Research Institute, Sovetskaya 13-15, Stavropol 355035, Russian Federation; d Anti-Plague Institute, M. Aikimbayev's Kazakh Science Centre for Quarantine and Zoonotic Diseases, 14 Kapalskaya Street, Almaty 050074, Kazakhstan; e RMIT University, School of Mathematical and Geospatial Sciences, Melbourne, Victoria 3000, Australia; f University of Liverpool, Institute of Integrative Biology, Crown Street, Liverpool, UK; g University of Antwerp, Department of Biology, Groenenborgerlaan 171, B-2020 Antwerpen, Belgium

**Keywords:** Object-based image analysis, Stratification, Landscape epidemiology, Vector-borne disease, Zoonosis, Yersinia pestis, Great gerbil, Desert environment

## Abstract

Plague is a zoonotic infectious disease present in great gerbil populations in Kazakhstan. Infectious disease dynamics are influenced by the spatial distribution of the carriers (hosts) of the disease. The great gerbil, the main host in our study area, lives in burrows, which can be recognized on high resolution satellite imagery. In this study, using earth observation data at various spatial scales, we map the spatial distribution of burrows in a semi-desert landscape.

The study area consists of various landscape types. To evaluate whether identification of burrows by classification is possible in these landscape types, the study area was subdivided into eight landscape units, on the basis of Landsat 7 ETM+ derived Tasselled Cap Greenness and Brightness, and SRTM derived standard deviation in elevation.

In the field, 904 burrows were mapped. Using two segmented 2.5 m resolution SPOT-5 XS satellite scenes, reference object sets were created. Random Forests were built for both SPOT scenes and used to classify the images. Additionally, a stratified classification was carried out, by building separate Random Forests per landscape unit.

Burrows were successfully classified in all landscape units. In the ‘steppe on floodplain’ areas, classification worked best: producer's and user's accuracy in those areas reached 88% and 100%, respectively. In the ‘floodplain’ areas with a more heterogeneous vegetation cover, classification worked least well; there, accuracies were 86 and 58% respectively. Stratified classification improved the results in all landscape units where comparison was possible (four), increasing kappa coefficients by 13, 10, 9 and 1%, respectively.

In this study, an innovative stratification method using high- and medium resolution imagery was applied in order to map host distribution on a large spatial scale. The burrow maps we developed will help to detect changes in the distribution of great gerbil populations and, moreover, serve as a unique empirical data set which can be used as input for epidemiological plague models. This is an important step in understanding the dynamics of plague.

## Highlights

► Local topography, Tasselled Cap Greenness and Tasselled Cap Brightness were used to create a landscape map using object-based analysis. ► A multi-scale object-based stratification approach improved accuracy of classification of burrows. ► The burrow maps provide realistic patterns to study the spread and persistence of plague.

## 1 Introduction

Plague, a disease mainly spread by rodents, was responsible for the deaths of nearly one-third of the European human population during a pandemic in the Middle Ages (Gage and Kosoy, 2005; Haensch et al., 2010). Plague is caused by the bacterium *Yersinia pestis* and can infect over 200 mammal species (‘hosts’), such as prairie dogs in the United States (Collinge et al., 2005), black rats in Madagascar (Keeling and Gilligan, 2000) and great gerbils in Kazakhstan (Davis et al., 2004). Plague is a vector-borne disease, i.e. the disease is transferred from host to host by a vector, fleas in the case of plague. In the last decades, plague, along with several other vector borne-diseases, has been resurging (Gubler, 1998). It causes about 2000 diagnosed human cases annually, of which the majority occur in Africa (World Health Organization, 2005). The disease is still considered dangerous; it is one of three diseases for which notification to the WHO is obligatory (World Health Organization, 1983).

Much of our knowledge of disease dynamics in natural populations is gained from investigations using epidemiological models (Hudson et al., 2001). For example, population size or density often must surpass a certain threshold in order for a disease to invade and/or persist (Lloyd-Smith et al., 2005), and higher population density typically increases the transmission rate (Begon et al., 2002). Moreover, the spatial distribution of hosts may determine local host connectivity, which when increased enhances the likelihood of successful disease spread (Keeling, 1999). When host distributions are patchy, increasing ‘patchiness’ may either increase or decrease the chance that an endemic infectious disease will persist (Hagenaars et al., 2004; Keeling and Gilligan, 2000; Jesse and Heesterbeek, 2011; Park et al., 2002). Thus, model studies suggest that the spatial distribution of hosts influences disease dynamics in different ways, but empirical data on the spatial distribution of infected and susceptible hosts on a population scale is scarce.

The distribution of animals is increasingly mapped by earth observation and remote devices on several spatial scales. On small scales, GPS-receivers are being used to track the animals. On a larger scale, remote sensing is used: several examples exist of host-habitat mapping (Bogh et al., 2007; Kalluri et al., 2007; Estrada-Peña, 2003). Such remote sensing based maps provide useful information on the characteristics of a focus (i.e. an area where the disease causing agent and its associated vector and/or host are present). As a consequence of rapid technological advancements in recent years, high spatial resolution imagery now offers the opportunity to map actual animal distributions, provided that a feature associated with the animal, like a burrow, can be identified on satellite images.

Burrows are constructed and used by certain rodents for sleeping, nesting and food storage. Burrowing rodents may diminish vegetation cover on and around their burrows such that it becomes possible to see them on satellite images, as is the case for wombats (*Vombatus ursinus*) in Australia (Löffler and Margules, 1980) and great gerbils (*Rhombomys opimus*) in Central Asia (Addink et al., 2010).

Great gerbils, social rodents that are numerous in (semi-)deserts in Central Asia, are important hosts of zoonotic infectious diseases, such as tularemia, cutaneous leishmaniasis and bubonic plague (Gage and Kosoy, 2005; Yaghoobi-Ershadi and Javadian, 1996; Atshabar et al., 2010). Plague in the great gerbil has been monitored extensively in Kazakhstan since the 1940s, although budget cuts since the 1990s have led to a severe decrease in plague surveillance (Gage and Kosoy, 2005; Ouagrham-Gormley et al., 2008). From this monitoring, we know that plague is endemic in 20 foci in Kazakhstan, the majority being steppe and desert foci (Atshabar et al., 2010). In each of these foci, plague monitoring, i.e. testing of rodents and fleas for plague, has been carried out under the supervision of Anti-Plague Stations. The finest spatial scale on which monitoring is carried out is the so-called sector, an area, defined by a latitude-longitude grid, of approximately 9.3 km × 9.8 km.

The frequent plague epizootics (epidemics in animal populations) do not result in massive die-offs of great gerbils, because the great gerbils are – in general – quite resistant to the disease (Gage and Kosoy, 2005; Biggins and Kosoy, 2001). The occurrence of these epizootics is related to the abundance of both host and vector. A host abundance threshold was first discovered by Davis et al. (2004) where abundance was measured as the percentage of burrows occupied by family groups. The plague threshold model was later improved by including the abundance of fleas (Reijniers et al., 2012). In both models burrow (density) maps form an important starting point for the prediction of plague.

In a pilot study by Addink et al. (2010), great gerbil burrows were successfully identified in an area of 6 km × 10 km using a Quickbird image with a spatial resolution of 2.4 m. Although this study offered a convincing proof-of-concept, burrows were only mapped in one landscape type. As the distribution of great gerbils is related to the landscape by food availability, local climate and competition with other species, the challenge is to map the great gerbil abundance across different landscapes. Moreover, the area investigated in the pilot study was smaller than the area of smallest scale plague monitoring by the Anti-Plague stations. Therefore, the relation between plague dynamics on the one hand and host abundance and structure on the other cannot be studied using these data. Mapping the great gerbil burrows over large areas, covering several plague monitoring units, and across several landscape types, will offer the opportunity to study the relation between landscape, great gerbil distribution and plague occurrence.

This paper focuses therefore on classifying burrows in two areas, of 60 km × 60 km and 60 km × 85 km, covering landscape types of fluvial and aeolian origin. The objectives are:

(1) To identify great gerbil burrows by semi-automated classification on high resolution SPOT-5 XS images across different landscape types and evaluate the accuracy.
(2) To construct a map of landscape units representing the variability and spatial structure of the landscape in this plague focus.
(3) To stratify the SPOT-5 XS images on the basis of these landscape units, and subsequently classify burrows per landscape unit, using local training data.

The methodology used to map the spatial distribution of burrows was as follows: landscape units were created on the basis of Landsat 7 ETM+ and SRTM data, using object-based analysis ($4.1). Then, the SPOT images were segmented and the burrows were classified, based on unstratified and stratified Random Forests ($4.2). Finally, the accuracy for both approaches was calculated ($4.3).

## 2 Study area

**Fig. 1:**
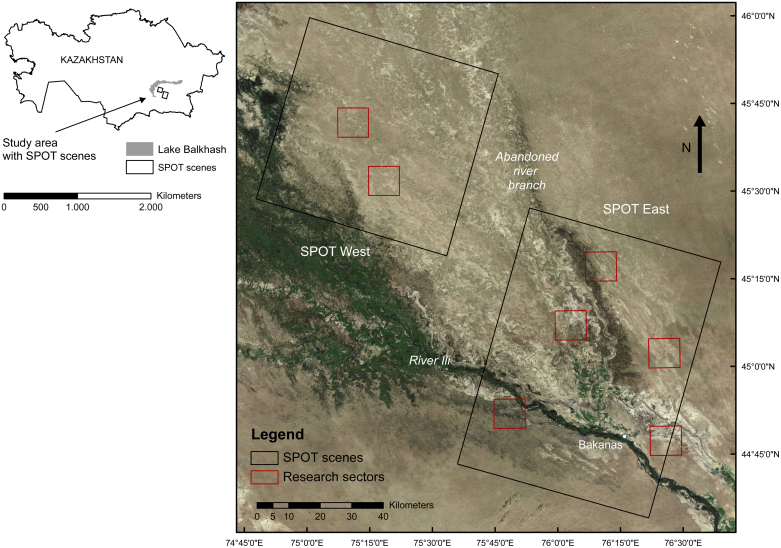
The study area in Kazakhstan south of Lake Balkhash (left), showing the Ili river delta, the location of the SPOT satellite scenes and the research sectors (right). Source: Bing maps, 2011.

The study area is a plague focus located in Eastern Kazakhstan, south of Lake Balkhash (Fig. 1), composed of fluvial and aeolian deposits. The area measures approximately 250 km × 200 km and encompasses a delta system developed by the Ili River. As the course of the Ili River has shifted over time from NNW to NW, several abandoned river branches can still be recognized in the landscape (Fig. 1). Large dune fields have formed along and on top of the abandoned floodplain. The irrigated agricultural area north of the town Bakanas was masked out.

Soils are sandy, with variable clay and low organic matter content: in the abandoned river beds gravel to sandy material is found, while further from the river branches, soils are more clay-rich or have dunes formed on top. In some areas soils are highly saline. The climate in the study area is strongly continental. Mean temperatures fluctuate dramatically, daily and within seasons, ranging between −40 °C in winter to +40 °C in summer (Suslov, 1961). Precipitation is less than 200 mm per year and falls primarily in winter (as snow) and spring (Suslov, 1961; Propastin, 2008). In spring snow melts quickly and forms small lakes (called takirs) in topographic depressions (Laity, 2008). Once dried up, these takirs are recognized easily because of their high albedo. Vegetation cover is variable, but shows, as well as a relation to soil moisture content, a declining trend in the direction of Lake Balkhash and with distance from the Ili River, with vegetation gradually changing from larger shrubs and reed grass thickets to lower shrubs and ephemeral grasses. Close to large abandoned river branches, vegetation cover is higher, most likely due to subsurface flow. In several areas, the soil is covered by a layer of lichen and moss. Overall, plant diversity and cover is low due to the yearly water deficit and extreme temperatures.

### 2.1 Great gerbils and their burrows

In this sandy (semi-)desert in the Balkhash basin, the great gerbil is the primary reservoir host for plague (Atshabar et al., 2010). Great gerbils are diurnal, mainly folivorous rodents that are well adapted to the climate. They live in family groups usually consisting of one male, one or more females and their offspring, and occupy one burrow per family (Randall et al., 2005). The rodents have a territorial nature and spend 90% of their activity within the boundaries of their own burrow (Rogovin et al., 2003), although visits to other burrows do occur (Rothshild, 1978). Because the numbers of gerbils fluctuate greatly, both inter- and intra-annually, burrows are not always occupied continuously: the percentage of occupied burrows is referred to as ‘occupancy’. During winter, occupancy usually drops because great gerbil mortality increases due to the harsh meteorological conditions. Also, because great gerbil movements are restricted, no new burrows are created during this period.

**Fig. 2:**
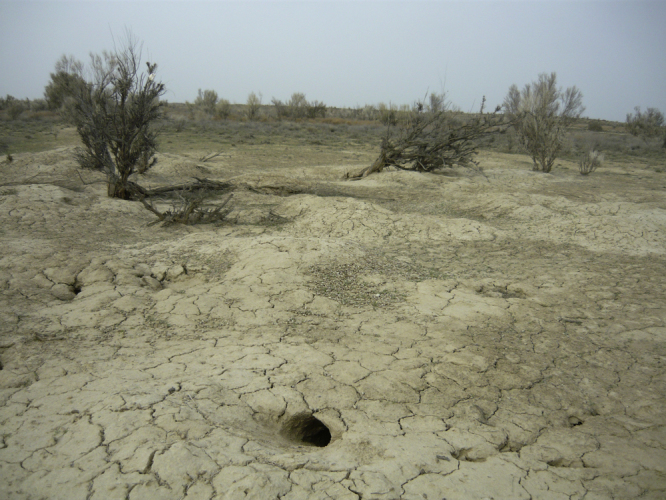
Burrow in alluvial material with a crusted soil. Multiple entrances, belonging to the same burrow, are visible. Black saxaul trees (*Haloxylon ammodendron*), visible in the background, form an important food source for the great gerbil.

The burrows of the great gerbil (Fig. 2) are omnipresent in the landscape and can be considered as relatively permanent, static features (Naumov and Lobachev, 1975). Burrows have multiple entrances that lead to a network of subterranean tunnels and chambers. They vary in size and shape in different landscapes (Naumov and Lobachev, 1975). Along dunes, burrows tend to be more elongated; on flat sandy substrates, burrows are circular, and in clay-rich soil they are more compact and deeper. Deeper burrows will lead to a more stable temperature inside the burrow and hence increase survival of the gerbils. Depending on the stability of the soil, a burrow can extend to 3 m deep (Naumov and Lobachev, 1975). All burrows have an ‘ecological centre’, i.e. the location of most intense activity which can be recognized by a high density of entrances. On the surface of a burrow, no or relatively little vegetation is present. This leads to an increase in albedo and hence enables the recognition of burrows on satellite images.

## 3 Data preparation

### 3.1 Field data

Data on burrow and non-burrow area locations were collected during field campaigns in September 2010 and April 2011, in seven sectors (Fig. 1). In these sectors 54 squares of 200 m were laid out as reference areas. Per sector, two or three locations were randomly selected (as far as accessibility allowed, because roads are scarce), so that variability within the sector would be covered. At each location, two or three squares were laid out, always a minimum distance of 200 m apart from each other.

The squares were then systematically checked for burrows of the great gerbil. Burrows, 904 in total, were numbered and marked. With a GPS navigation device, the burrows were mapped by recording the coordinates at the ecological centre. In addition, burrow diameters were measured.

### 3.2 Satellite images

Three pan-sharpened 2.5 m resolution orthorectified SPOT-5 images containing green, red and NIR spectral bands were acquired for October 2010 (Astrium, 2012). They cover two areas of 60 km × 85 km in the east (SPOT East) and 60 km × 60 km in the west (SPOT West) and overlap the seven research sectors (Fig. 1). SPOT West was acquired on 14 October and the two images composing SPOT East were acquired on the 9 October.

Landsat 7 ETM+ images (28.5 m resolution) from 2000 and 2001 were used for the landscape stratification together with a 90 m resolution Digital Elevation Model (DEM) acquired by the Shuttle Radar Topography Mission (SRTM). Both Landsat and SPOT images were converted to top-of-atmosphere reflectance (Chander et al., 2009).

## 4 Methods

**Fig. 3:**
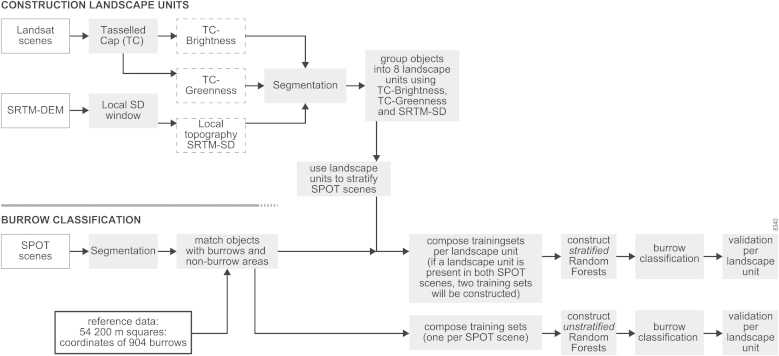
Flowchart of the sequential processing steps of the construction of the landscape units (top) and the burrow classification (bottom).

An overview of the processing steps is given in Fig. 3.

### 4.1 Defining landscape units

In order to construct landscape units in the Balkhash basin, first the most important variables that determine the variability of landscapes in this area were listed. These variables are soil type, vegetation cover and local topography. These landscape variables also determine to a large extent the physical habitat suitability for the great gerbil. Soil type influences the possibilities of quality burrow construction, as the cohesion of the soil determines whether it is possible to construct a deep and stable burrow. Vegetation cover reflects the presence of food resources. Local topography influences soil depth, as depositional and erosional processes are influenced by topography. Also, along with vegetation cover, it has influence on the ability to hide from predators, like birds of prey. A combination of these three variables will represent different local great gerbil habitats and in addition will likely influence the ability to detect the burrows in satellite images.

The landscape units were defined in three steps (see also Fig. 3):

(1) Landscape representative layers were calculated using Landsat 7 ETM+ scenes and the SRTM-DEM. To represent the variation in soil types, a soil Brightness layer was derived from the Landsat images using the Tasselled Cap Brightness (TC-Brightness) (Crist and Cicone, 1984). TC-Brightness is a measure of overall reflectance and makes it possible to differentiate light soils from darks soils. The variation in vegetation cover in the area was extracted from the Tasselled Cap Greenness (TC-Greenness) (Crist and Cicone, 1984). TC-Greenness displays the contrast between the near-infrared reflectance and the visible reflectance and is a measure of the presence and density of green vegetation. The local variation in topography was computed using the SRTM-DEM, specifically the standard deviation of the SRTM-DEM, calculated using a window size of 3 by 3 pixels. The pixels of the local SRTM-DEM standard deviation (DEM-SD) were then matched to 28.5 m pixels using nearest neighbour resampling.
(2) Multi-resolution segmentation was applied to these three layers (giving each layer an equal weight), using eCognition^®^ Developer Software (Trimble, 2011). Segmentation is the process of grouping neighbouring pixels on the basis of a homogeneity criterion (Blaschke and Strobl, 2001). This homogeneity criterion (i.e. the scale parameter) was chosen such that the objects were large enough to meaningfully represent a landscape type, but small enough to avoid grouping of different landscape types into one object. This resulted in a median object size of 6.9 km^2^.
(3) These objects were then classified into different landscape units. Classification of the objects was essential, because objects with a similar landscape type do not necessarily border each other, which means they would not have been grouped together during segmentation. To classify each object inside the two SPOT areas, its mean values of TC-Greenness, TC-Brightness, and DEM-SD were obtained, to form three object attribute layers. The median value of each layer was then used to separate it into high and low classes (above and below the median). These high and low classes for the three variables were then combined (2 × 2 × 2) to form eight landscape units with either a high or a low value for TC-Brightness, TC-Greenness, and DEM-SD, which we denote by a capital and lower case letters for a high and low values, respectively (B, b, G, g and D, d). Finally, landscape units were given a geomorphological and landscape-ecological interpretation and description, using field observations and literature on the area (Suslov, 1961; Laity, 2008).

### 4.2 Burrow classification

#### 4.2.1 Creating a reference object data set: combining field and satellite data

First, SPOT images were subjected to an edge-preserving smoothing filter (Nagao and Matsuyama, 1979) in order to improve object stability (Addink, 2012). They were then segmented using tiling and stitching, to keep the segmentation process fast and efficient. The three bands were given equal weights and the shape parameter was set to 0, which means that pixels are grouped based on their reflectance values only (and not on the shape of the resulting object) (Baatz and Schäpe, 2000). Segmentation was carried out on such a scale that object size coincided with the size of burrows, resulting in a mean object size of 23.8 m^2^. In total, in the western and eastern 9,659,972 and 12,868,017 objects were created respectively.

The objects resulting from the segmentation have associated attributes that were calculated using eCognition^®^. These attributes – also called independent variables – give information on the spectral-, texture-, shape-, size- and neighbour-properties of the objects. For example, the variable *Mean NIR* gives the mean reflection in the NIR band for an object and the variable *Number of darker objects* gives the number of darker objects surrounding the specific object. In total, 67 independent variables were calculated, of which 42% consisted of spectral- and texture-, 31% of neighbour- and 27% of shape- and size variables.

The objects and associated independent variables were used to create reference object sets (one for each SPOT scene), containing burrow and non-burrow area objects. Using the method from Addink et al. (2010), objects representing burrows were selected in two steps. First, all objects within the estimated position error of a burrow field location, as given by the GPS, were marked as potential burrow objects. Second, from those potential burrow objects, for each field observation, the brightest object was selected. This is calculated by taking the average of all pixel values in an object (so-called *Brightness* - not to be confused with the TC-Brightness mentioned in $4.1). Non-burrow area objects were defined as the objects that were not marked as a potential burrow object and were located completely within the 200 m research squares. The non-burrow area objects include therefore a range of local landscape features, such as a patch of shrubs or grass, a (part of a) dune, a takir or a road.

The reference object data set for SPOT West then consisted of 315 burrow objects and 787 non-burrow area objects, and for SPOT East these numbers were 589 and 2311, respectively.

#### 4.2.2 Building Random Forests

Random Forest is a robust statistical classifier that makes a prediction of a test set based on observations from a training set, using multiple decision trees (Breiman, 2001). Using the values of the independent variables, each tree consists of nodes where the training set is split into homogenous subsets of burrow and non-burrow areas until the tree is fully grown, i.e. until the whole training set is subdivided into those classes. The Random Forests used in this study were created with the *randomForest* package (Liaw and Wiener, 2002) implemented in the R statistical programming environment (R Development Core Team, 2011).

Every tree was constructed using a 2/3 bootstrap (default value) of the training set. The 1/3 bootstrap not used to calibrate the tree, the so-called “Out-Of-Bag” (OOB) data, are, by running these samples through the Random Forest, used to derive an error estimate (“OOB-error”). The average value of the OOB-errors for all the trees gives a good indication of how well classes can be separated (Rodriguez-Galiano et al., 2012b).

A Random Forest chooses for each node on a tree the best predictive variable from a random subset (with the size of the square root of the total number of independent variables) of the predictor variables. This means that if there are many variables with little predictive power, the Random Forest might be sub-optimal. Therefore, it is useful to select the most predictive variables in advance of building the final Random Forest (Rodriguez-Galiano et al., 2012a). The Random Forest classifier itself contains a novel method to estimate variable importance (Verikas et al., 2011; Cutler et al., 2007), which can be used to exclude the least predictive variables in advance. By running the Random Forest iteratively and excluding the least predictive variables at every run, the most parsimonious solution can be found that has the least variables amongst those within the sampling error from the solution with the absolute smallest OOB-error (Diaz-Uriarte, 2007).

The Random Forests built in this study used 10,000 trees, which is a sufficient amount of trees to ensure the OOB-error is stable; for other Random Forest settings the default values were used. All Random Forests were optimized by removing the least predictive variables using the *VarSelRF* R package (Diaz-Uriarte, 2007).

#### 4.2.3 Unstratified classification

Classification using Random Forest is done by running all observations, in this case objects with associated variables, along each tree, so that each object gets multiple, votes (in this case 10,000). The majority of all the votes decide whether burrow or non-burrow area is assigned to a specific object (Liaw and Wiener, 2002). This is then repeated for all the objects.

One Random Forest was constructed per SPOT scene to classify the burrows. Constructing one Random Forest for both SPOT West and East was not desirable, because the SPOT images were acquired on different dates with slightly different atmospheric conditions. The Random Forests were trained with training sets that contained equal numbers of burrow and non-burrow area objects, since imbalanced training sets considerably influence the outcome of the Random Forest classification (Stumpf and Kerle, 2011). 80% of the burrows were used for training, 20% was used for validation. For example, in the eastern Image 470 burrows (=80% of 587) were selected by drawing a random sample. Then, in the same manner, 470 non-burrow area objects were selected. Together they formed the unstratified training set for the eastern SPOT image.

#### 4.2.4 Stratified classification: classification per landscape unit

Stratification is the process of using ancillary information to divide the investigated area spatially into units (strata), so that they can be analyzed separately (Vintrou et al., 2012; Hornstra et al., 2000). Stratification prior to classification allows an optimal classification procedure for specific conditions in different areas. In our research, stratification was used because of the hypothesis that the ability to identify burrows would be differentially affected by relationship between a burrow and its surroundings in different landscapes. For example, in the sparsely vegetated, inactive floodplains in the study area, burrows are better recognizable because the “background”, i.e. non-burrow area, is relatively homogenous. In the areas closer to (abandoned) river beds, where water availability leads to more variation in vegetation cover and type, the burrows are less distinct against their background.

To stratify the SPOT scenes, the obtained landscape units define the borders of the strata, i.e. instead of building one Random Forest per SPOT scene, Random Forests are built per landscape unit. Stratified Random Forests can only be built when sufficient field observations are available, which is the case for four landscape units (Table 1), namely bgd (East), Bgd (West and East), bGd (East) and BgD (West and East). For each of these six units stratified Random Forests were built (Table 1). The stratified Random Forests were–like the unstratified Random Forests–trained with balanced training sets, using burrows and non-burrow areas located in the corresponding landscape unit.

#### 4.2.5 Refining the Random Forest classification

Due to the territorial nature of great gerbil families, burrows are always at a certain minimum distance from each other, i.e. they have an inhibited pattern. This information was used to improve the classification following the procedure of Addink et al. (2010): if a burrow was adjacent to other classified burrow objects it would remain classified as a burrow when the *Brightness* of adjacent burrow objects was lower, otherwise it would be reclassified into a non-burrow area. This ensured that large burrows consisting of two objects would only be counted as one burrow.

### 4.3 Validation

Random Forests were created using 80% of the burrow field data and the remaining 20% was used for validation. For validation it is important to have a representative validation data set (Congalton, 1991). The 20% validation set was constructed in such a way that the ratio of burrows to non-burrow areas equalled the ratio found in the field.

Validation was done by calculating the producer's, user's and overall accuracy (Lillesand et al., 2004) based on counts for every landscape unit, using the burrow locations buffered with their diameter. Although landscape units Bgd and BgD are present in both SPOT scenes, validation of landscape units Bgd and BgD was done in the same manner. The kappa coefficient, a measure of overall accuracy based on the classification error matrix, was also calculated for each unit (Congalton, 1991). To compare the classification accuracies for different landscape units, the values of the producer's, user's and overall accuracy, as well as the kappa coefficient were compared. To evaluate the difference between the unstratified and stratified method, additionally, significance was tested at the 95% confidence level using the Kappa Analysis Test of Significance (Congalton et al., 1983; Congalton, 1991).

Moreover, based on the field data, the densities of burrows in the research squares were calculated, by dividing the number of burrows inside the respective squares by the area covered by the squares. Then, the ratio between the density predicted by the classification and the density observed in the field was calculated, to get an estimate of the accuracy of the density of the created burrow maps.

## 5 Results

### 5.1 Landscape units

**Fig. 4:**
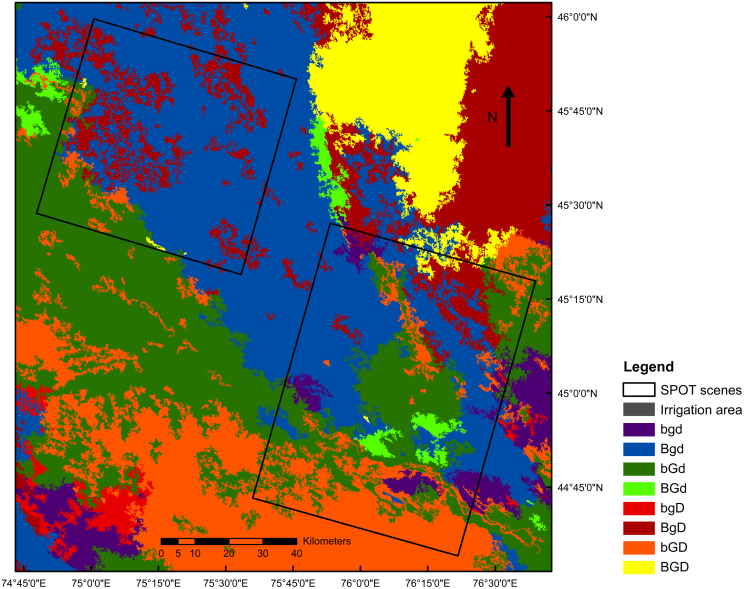
Landscape units identified in the Balkhash basin based on high and low values of TC-Brightness (B/b), TC-Greenness (G/b) and local variation in topography (D/d). The description of the units is given in Table 2.

Eight landscape units were distinguished in the Balkhash basin resulting from the landscape stratification using the layers TC-Brightness, TC-Greenness and DEM-SD (Table 2 and Fig. 4). Four units describe the floodplain deposits: steppe on inactive floodplain (bgd), steppe on inactive floodplain with salt flats (Bgd), active floodplain with shrub dominated vegetation (bGd) and floodplain with developing vegetation (BGd). The other four units describe the dunes: dynamic dunes (BgD), stabilized dunes with inactive vegetation (bgD), stabilized dunes, with active vegetation on moist soil (bGD) and stabilized dunes with active vegetation (BGD).

**Fig. 5:**
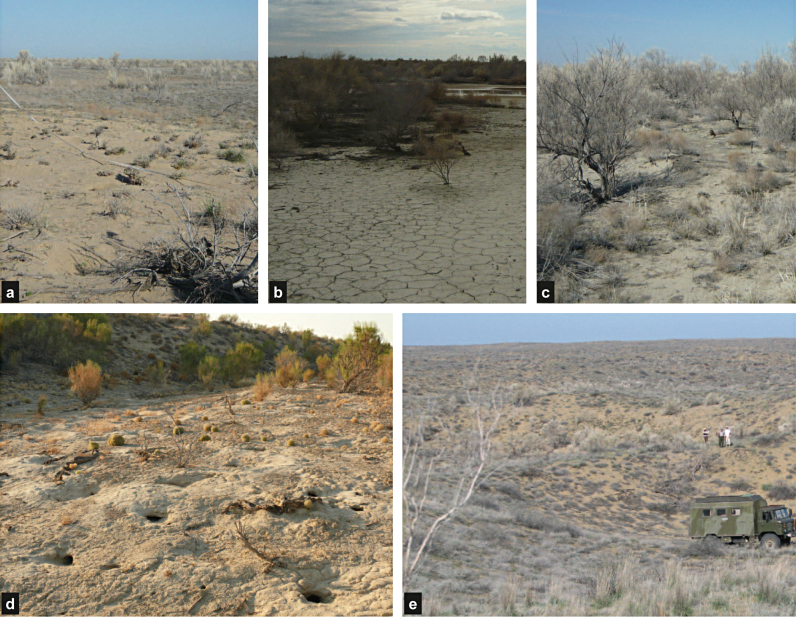
Photographs of the different landscape units. (a) Unit bgd, steppe on inactive floodplain. In the front a burrow is measured. The area is flat, formed by fluvial deposits and has little vegetation. (b) Unit Bgd, steppe on inactive floodplain with salt flats. In the background a lake formed by the snow melt is displayed. (c) Unit bGd, active floodplain with shrub dominated vegetation. (d) Unit BgD, dynamic dunes (BgD), with an occupied burrow in the front. (e) Unit bGD, stabilized dunes with active vegetation on moist soil. Here vegetation consists of grasses rather than shrubs.

The steppe on inactive floodplain (bgd) is a unit where a homogenous low vegetation cover formed on a very fine sandy-silty soil (Fig. 5a). The steppe on inactive floodplain with salt flats (Bgd) also has a low vegetation cover on mostly silt to clay-rich soils. Abundant takirs have formed in small depressions in the landscape (Fig. 5b). Unit bGd, the active floodplain with shrub dominated vegetation, covers most of the current Ili delta and part of the abandoned Ili river branch. In this unit vegetation cover is more abundant and consists mostly of 2–3 m high shrubs (Fig. 5c). The floodplain with developing vegetation (BGd) is not prominently present in the study area. It consists of chlorophyll rich and active vegetation such as reed grasses formed in small depressions close to the Ili River. The dynamic dunes (BgD) are low dunes (<10 m) with a low cover of grasses (Fig. 5d). The stabilized dunes with inactive vegetation (bgD) covers only a small area of the study area and is typified by a low and inactive vegetation cover. The stabilized dunes with active vegetation on moist soil (bGD) is present in the south and close to the Ili River. These dunes are higher compared to the dynamic dunes and have vegetation that consists of both grasses and low shrubs (Fig. 5e). The stabilized dunes with active vegetation (BGD) is comparable to the previous unit (bGD) but occurs in the north where less water is available, which results in a higher value in TC-Brightness.

In general, TC-Brightness, a measure of overall reflectance in the Landsat bands, shows an increase from south to north, and is lowest in areas with a high water content, such as in the active floodplain with shrub-dominated vegetation (bGd). TC-Greenness shows an opposite pattern and decreases towards Lake Balkhash, while it is highest in the Ili River delta.

All the eight landscape units are present in the SPOT satellite scenes (Table 2), although the stabilized dunes with inactive vegetation unit (bgD) only covers an area of about 4 km^2^. The steppe unit on inactive floodplain with salt flats (Bgd) is most abundant and represents 47.5% of the surface area in SPOT West and East. Interestingly, the floodplain with developing vegetation unit (BGd) also appears north of SPOT East while there is not an active river present; this is presumably because of subsurface flow towards Lake Balkhash through the abandoned river branch, which enables more growth of vegetation.

**Fig. 6:**
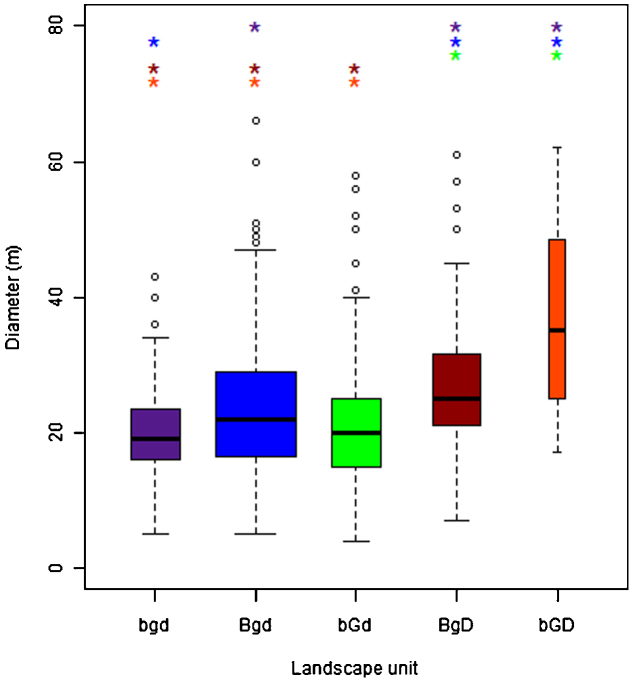
Burrow diameters per landscape unit (colours correspond with colours in Fig. 4). The widths of the boxes are proportional to the sample size in each landscape unit. The colours of the asterisks (*) indicate with which landscape unit there is a significant difference in the mean (a = 0.05). The codes of the landscape units can be found in Table 2.

In five out of the seven units located in the area covered by the SPOT scenes, field work data are available (Table 2). Diameters of each burrow were measured in the field (Fig. 6). Burrow diameters range from a mean of 21 m (minimum 4 m) in the clay-rich soils in the active floodplain (bGd) to a mean of 37 m (maximum 62 m) in the stabilized dunes in the southeast (bGD) (Fig. 6). Burrow diameters smaller than 10 m often correspond to old and abandoned burrows. The largest diameters are found in the dunes, where the burrows are elongated and situated in line with the dunes.

The variance of burrow diameters is largest in unit bGd and differs significantly from the variances in the other units (*F*-test of the equality of variances using the natural logarithm of the diameters, at *α* = 0.05). The high diameter-burrows in this unit (see outliers in unit bGd, Fig. 6) are almost all found in two squares, which are located on the border with unit bGD. This explains the relatively high variance in this unit. When these squares are removed from the analysis, the variance of unit bGd is only significantly different from the variance in unit BgD, but equal to the other units. The variance in burrow diameters is lowest in the steppe on inactive floodplain unit (bgd), which reflects the landscape homogeneity in this unit.

The mean diameters of the burrow diameters per landscape unit differ significantly from each other, except for bgd-bGd, Bgd-bGd and BgD-bGD (Fig. 6), which was tested pair-wise using *t*-tests with Bonferroni adjustment, at *α* = 0.05, on the natural logarithm of the diameters.

Burrows located in fluvial deposits are on average smaller than burrows in aeolian deposits. This result is not surprising, as in clay-rich fluvial soils burrows tend to be more compact and deep, whereas in sandy aeolian soils burrows are larger, but less deep (Naumov and Lobachev, 1975).

### 5.2 Unstratified classification

**Fig. 7:**
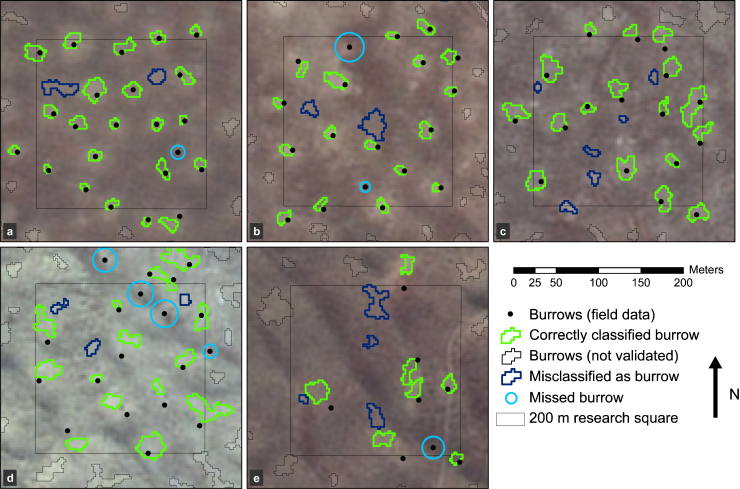
Maps showing examples of classification results in different landscape units. The results are located in (a) unit bgd; (b) unit Bgd; (c) unit bGd; (d) unit BgD and (e) bGD. The codes can be found in Table 2; the location of the landscape units in the area can be found in Fig. 4. Of the squares displayed, bgd, Bgd, bGd and bGD are located in SPOT East; the BgD square displayed is located in SPOT West.

In this section we describe the results of the unstratified classification of burrows, which we evaluate per landscape unit. In Fig. 7 examples are given of classification results in the five landscape units where burrow reference data are available. These maps show that burrow classification is successful across different landscape units, in both SPOT images, although with varying accuracies. For example, in unit bgd (Fig. 7a), 22 of 23 burrows are correctly identified. In unit bGd (Fig. 7c) it can be seen that small takirs are often misclassified as burrow. In the displayed squares of the dunes (Fig. 7d and e), 22 out of 27 burrows are recognized. Burrows located in the shadow side of a dune are often not recognized and especially in bGD there is an overestimation of burrows.

For each landscape unit the producer's, user's and overall accuracies were calculated, as well as kappa coefficients of agreement and the accuracy of the predicted burrow density (Table 3). Accuracies and kappa coefficients are in general high but nonetheless vary considerably between landscape units. Classification of burrows works extremely well in the steppe on inactive floodplain (bgd), where producer's and user's accuracies reach 88% and 92% respectively. Classification works least well in the active floodplain with shrub dominated vegetation (bGd), where the user's accuracy is only 48%. In most squares in this unit burrows are overestimated, but there is a considerable variation in accuracy between the squares. Classification in the dynamic dunes (BgD) works remarkably well: producer's and user's accuracies amount 84% and 77%. In the stabilized dunes with active vegetation on moist soil (bGD) classification accuracies are also quite high (producer's and user's accuracy of 100 and 60% respectively) and indicate classification of burrows is also possible in this more complex landscape unit. It should however be noted that only few validation data were available in this unit (Table 1) and therefore the producer's accuracy for this unit is likely to be overestimated.

The ratio of predicted burrow density to observed burrow density shows how well burrow density can be predicted: values range from 0.86 to 1.75. In areas with high TC-Greenness (G) burrows are overestimated. In unit bGd the overestimation is largest. This is partly due to the occurrence of a large number of takirs in certain field squares, as takirs are easily misclassified as burrows. When three squares with a large number of takirs are removed from the density calculation for this unit, the ratio predicted to observed decreases to 1.5. For the total area, i.e. including the data of all landscape units, the ratio between predicted and observed burrow density is 1.16.

The unstratified Random Forests created to classify the SPOT scenes use a selection of the 67 independent spectral, shape and neighbour variables included in the initial reference object sets. Both the Random Forest algorithms used for the unstratified classification, i.e. one for the western and one for the eastern scene, used mostly neighbour values (78% West and 53% East) such as *Mean difference to neighbours* and *Relative border to brighter neighbours*. Spectral and textural variables amounted 22% in the Western Random Forest and 35% in the Eastern. Least selected were variables indicating the shape of objects.

### 5.3 Stratified classification

For four landscape units, i.e. units bgd, Bgd, bGd and BgD, stratified Random Forests were created, to evaluate whether this would improve classification accuracies (bgD was left out because too few field data were available). The kappa coefficients and their variances (Congalton et al., 1983; Foody, 2004) for the unstratified and stratified classification were used to compare the performance of the unstratified and stratified approach (two-sided test of kappa coefficients, at *α* = 0.05).

Stratified classification results in significantly higher kappa coefficients in all four landscapes units (Table 3). The improvement due to stratification is clearest in unit bgd, where four out of five metrics improve and one remains the same when stratified classification is used. For unit BgD the improvement is visible in four out of five metrics; only the density is slightly less well predicted. In bGd however, only three metrics improve when using stratified classification; the producer's accuracy remains similar, while the ratio predicted to observed density shows there is an increased overestimation of burrows. This overestimation occurred in squares with a large number of takirs. When three squares with a large number of takirs are removed from the calculation, the predicted accuracy improves to 1.35, i.e. then stratification does improve also the predicted accuracy. In Bgd stratification improved the kappa coefficient slightly. The predicted density in this unit using the stratified Random Forest is however almost completely accurate (0.99).

The stratified Random Forests each used a subset of variables that resulted in the lowest OOB-error. Between 3 and 27 of the independent 67 variables were selected for the six stratified Random Forests (Table 5). Of the 67 variables, 32 variables were never selected. These variables consisted of eighteen (56%) spectral variables, thirteen (40%) shape and one (3%) neighbour variable.

The majority of the variables selected in the Random Forests, like in the unstratified Random Forests, are neighbour variables; variables least selected are shape and size variables. Most selected variables are *Mean difference to neighbour Red* and *Relative border to brighter objects NIR* (Trimble, 2011); they were selected by five out of six stratified Random Forests. In total 25 variables were selected by two or more Random Forests; in other words, there was a considerable overlap in selected variables. No remarkable differences in selected variables, or in distribution between spectral, neighbour and shape variables, were found that could be related directly to characteristics of the specific landscape unit.

The variables selected by the stratified Random Forests were compared with the selected variables in the unstratified Random Forests. For five out of six stratified Random Forests, fewer variables were selected compared to the unstratified Random Forests, only Bgd (West) selected two variables more compared to the unstratified Western Random Forest. The variables selected by the unstratified and stratified Random Forest resembled considerably, for example, 26 out of 27 variables selected by the stratified Random Forest bgd (East) were also selected by the unstratified Eastern Random Forest and all 14 variables selected by stratified Random Forest bGd (East) were also selected by the unstratified Eastern Random Forest. In BgD (East) there was a difference; here, the relative contribution of shape variables increased compared to the unstratified Eastern Random Forest.

### 5.4 Burrow densities in the landscape units

**Fig. 8:**
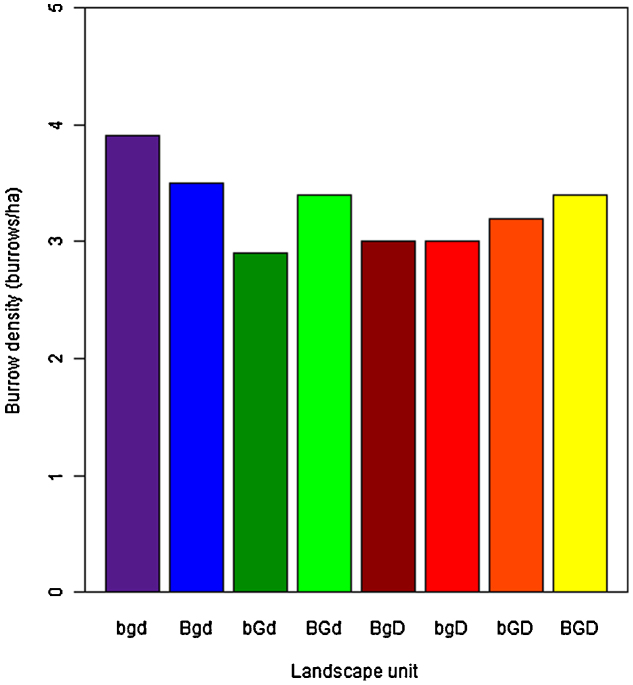
Burrow density per landscape unit (colours match with the units on the map in Fig. 4), calculated based on the burrow maps.

Using the results from unstratified classification for units BGd, bgD, bGD and BGD and the stratified classification for units bgd, Bgd, bGd and BgD the final burrow maps (West and East) were created. Mean burrow densities were calculated per landscape unit (Fig. 8), based on the entire units for the smaller units (bgd, BGd, bgD and BGD) and on a minimum area of 96 km^2^ for the larger units (Bgd, bGd, BgD and bGD). The mean burrow densities per landscape unit range from 2.9 burrows/ha in the active floodplain with shrub dominated vegetation (bGd) to 3.9 burrows/ha in the steppe on inactive floodplain unit (bgd). These burrow density results correspond to the range of burrow density estimates collected by the Anti-Plague stations which vary from 0.89 to 4.8 burrows/ha (Davis et al., 2008). On average, burrow densities are lower in the four aeolian units than in the floodplain units. On the floodplain, areas with high TC-Greenness (G) have lower burrow densities compared to areas with low TC-Greenness, whereas in the dunes, the opposite is true.

At a smaller scale, burrow density is more variable, as demonstrated by the variable numbers of field observations of burrows detected in the research squares: the lowest density of burrows was found in the vegetated floodplain and delta (bGd); the minimum was 0 burrows/ha. A maximum density of 6.25 burrows/ha was found in a research square located in the sparsely vegetated floodplain (Bgd).

### 5.5 Burrow maps for epidemiological modelling

**Fig. 9:**
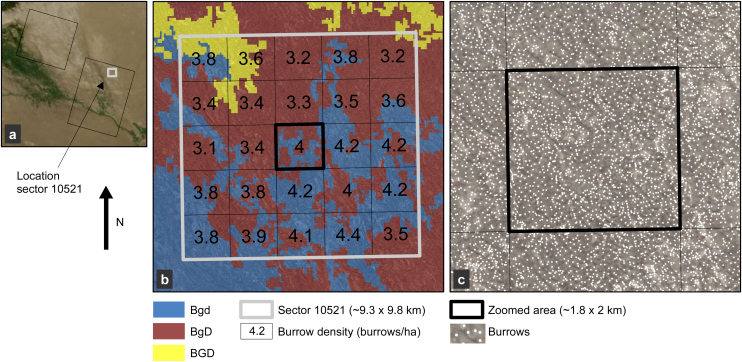
(a) The location of sector 10521. (b) Sector 10521 with its burrow densities calculated for 25 cells. (c) Zoom of sector 10521. Individual mapped burrows are shown.

An example of a density map of a sector is shown in Fig. 9. The densities are shown in squares of ∼1.8 km × 2 km (Fig. 9b). The mean density in this sector is 3.7 burrows/ha and varies between 3.1 and 4.4 burrows/ha. The Anti-Plague station estimated the density in this sector at 3.0 burrows/ha. Individual mapped burrows are shown in Fig. 9c.

The producer's accuracy was evaluated for occupied and empty burrows separately. Producer's accuracy was found to be 85% for occupied burrows and 74% for empty burrows. In other words, occupied burrows are more easily recognized than empty ones.

## 6 Discussion

### 6.1 Accuracy of the burrow maps

Remote sensing is increasingly used in epidemiological research on infectious and vector-borne diseases (Hay, 1997; Atkinson and Graham, 2006; Beck et al., 2000; Neerinckx et al., 2008). Commonly, remotely sensed derived variables are used to map habitat suitable for vectors (Tran et al., 2008; Kalluri et al., 2007; Hay and Lennon, 1999). In this study, remote sensing was used not only to map habitat suitable for hosts, but to map the exact distribution of the host's residences over various landscape types.

The great gerbil burrow maps were created by using a Random Forest classification algorithm on burrow and non-burrow area objects with associated variables created from high-resolution satellite imagery. This resulted in burrow maps with median producer's and user's accuracies of 86% (77–100%) and 81% (58–100%). The classification accuracies found in this study are, compared to the pilot study carried out by Addink et al. (2010), higher and more balanced. Producer's and user's accuracies for the present study, calculated for the study area of Addink et al., are 90% and 95% respectively. Producer's and user's accuracies found by Addink et al. are 85% and 62%, while their training set had more training data available. The higher classification accuracy found in this study is likely caused by two factors. Firstly, the Random Forest classifier is a more advanced and robust classification algorithm (Rodriguez-Galiano et al., 2012b; Duro et al., 2012) than the decision tree classifier which was used by Addink et al. (2010). Secondly, in this study, the non-burrow area selection was unbiased, which was possible because of the burrow field sampling in squares, whereas Addink et al. selected non-burrow areas in the field which is more prone to bias. The classification accuracies found in the current study are comparable to other object-based high-resolution studies that map relatively small landscape features in natural environments, such as the mapping of downed logs (Blanchard et al., 2011).

### 6.2 Validity of the burrow maps

The burrow maps are based on SPOT-5 images acquired in October 2010. The temporal validity of the maps is currently unknown, because detailed information on the lifetime and renewal rate of burrows is lacking. The lifetime of a burrow depends on various factors (Naumov and Lobachev, 1975). It is likely influenced by the occupancy time and on the time it takes to become re-occupied in case the burrow is abandoned. According to Naumov and Lobachev (1975), the lifetime of burrows can reach hundreds of years, but this may well depend on the type of soil in which they are constructed. As mentioned previously, burrows in more clay-rich material tend to be more solid and deep, whereas the burrows in sandy soil such as in the dunes are more shallow and elongated. As observed in the field, the time of disappearance of burrows, i.e. the time that elapses from the moment a burrow is abandoned until the burrow can be neither recognized nor re-colonized, seems to be affected by landscape type, although no detailed field data are yet available. As a result of the lower cohesion of the soil, the time of disappearance is likely to be shorter in the sandy soils in the dunes compared to the clay-rich soils in the floodplain. For the burrows maps created, it means that the period of validity for a map will be longer for the floodplain areas (landscape unit bgd, Bgd and bGd) than for areas with mostly sandy material (BgD, bGD and BGD). The question of the lifetime of burrows could be investigated by mapping the burrows using the method presented in this paper in subsequent years, and apply change-detection to analyse the differences and dynamics.

### 6.3 Classification per landscape unit versus unstratified classification

In advance of classification per landscape unit, landscape units were created based on TC-Brightness and TC-Greenness of Landsat scenes in combination with the SRTM-DEM derived local topographic variability (DEM-SD). This resulted in a map with a clear and realistic spatial pattern, corresponding with field observations. The use of Landsat imagery with its spatial resolution of 28.5 m offered the opportunity to map the landscape over large areas. Although the spatial resolution of the SRTM-DEM is lower than the resolution of Landsat, the SRTM-DEM still adds useful information for the landscape mapping. Using high resolution spatial imagery, such as SPOT-5 XS, for landscape mapping would not be as successful because the small pixel size would result in a too high variance to be able to map landscape units accurately. The combination of high and medium resolution imagery for burrow mapping across landscapes thus offers an effective method.

The classification per landscape unit resulted in considerably higher classification accuracies in three landscape units and in slightly higher accuracies in one landscape unit. Generally, stratification in combination with a Random Forest classifier might improve classification results because (1) the variable selection is fitted to the specific area, (2) the object training set used to train the Random Forest is selected from the specific area and hence the threshold values used in the tree to separate burrows from non-burrow areas are more likely to fit the objects in the specific area.

In the case of the Balkhash area, burrow diameters showed that significant differences per landscape unit exist in burrow size. Non-burrow areas also obviously differ per landscape unit, which was shown by the differences in values in TC-Brightness, TC-Greenness and SRTM DEM-SD.

**Fig. 10:**
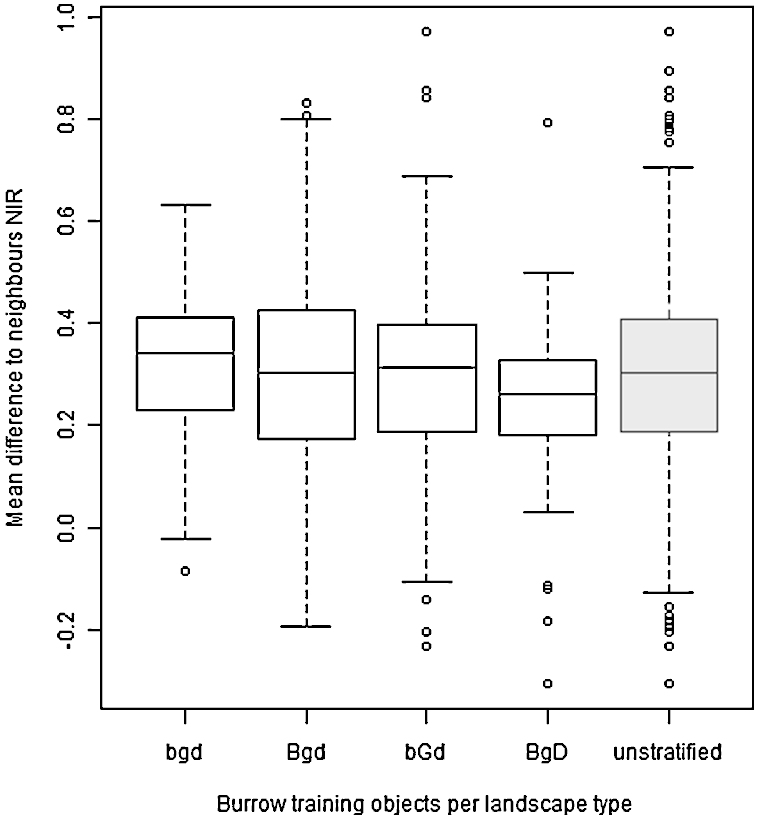
Boxplots of Mean difference to neighbours NIR in each landscape unit for burrow objects. The range differs per landscape unit.

In the three landscape units where stratification improved results considerably, fewer variables were selected compared to the unstratified classification. Also, the range for an often selected variable such as *Mean difference to neighbours NIR* is smaller for stratified training sets than for the unstratified training sets (Fig. 10), which might be an additional explanation for the success of the stratification. In unit Bgd stratification did not improve results much, but it did not decrease classification accuracies either. A possible explanation for this is that the variables associated with the burrow and non-burrow areas in the Bgd training set cover a (nearly) similarly large range as the unstratified training set (see for example Fig. 10), which means that stratification has less influence.

The underlying reason for classification results only improving slightly in Bgd is thus that the Bgd unit itself is more heterogeneous than the other units. This could be improved by splitting this area into two sub-units, for example based on TC-Brightness. However, stratified classification might perform less well if there is a lack of field data per landscape unit. On the other hand, when stratification creates only few landscape units, the units may be too heterogeneous, and consequently the training set will be less representative. Generally, for stratification it is therefore important to find a balance between the homogeneity of a landscape unit and the amount of field data for classification per unit. To achieve this, landscape stratification can be carried out in advance of the creation of a sampling scheme. After this, a thorough look at the ranges of variables might give an indication of whether stratification will improve results.

Because burrows vary in appearance and heterogeneity over different landscapes, the use of multiple scale parameters might further improve classification accuracy, because across landscape units, slightly different heterogeneity thresholds might be optimal (Anders et al., 2011). This could be of use for unit bGd, where both unstratified and stratified classification resulted in relatively low classification accuracy. In this unit, objects are often large compared to the burrow size, which means that training of the Random Forest, but also the classification itself performs less well. To improve the accuracies in this unit, the unit could be split into four subunits based on TC-Brightness and TC-Greenness, then separate scale parameters could be used in combination with more intensive ground truth collection.

### 6.4 Burrow density in the landscape units

The ratio of predicted and observed densities for the landscape units has shown that density can be predicted moderately to very accurately. In units with a high TC-Greenness (bGd and bGD) burrows are systematically overestimated, which can likely be attributed to two reasons. Firstly, the vegetation cover in units with a high TC-Greenness is often relatively heterogeneous, which means that areas with a lower vegetation cover are often incorrectly classified as burrow, because their difference to their neighbours resembles the difference between burrows and their neighbours. Secondly, in the areas with higher TC-Greenness vegetation often consists of shrubs, which are not always removed by the great gerbils from the surface of burrows. These burrows therefore do not have such a distinct signal. In areas with low TC-Greenness (bgd, Bgd and BgD) vegetation cover is much more homogeneous (compare for example Fig. 7b with c). Additionally, in some areas, especially in units bgd and Bgd, vegetation also consists of lichen, but is removed from the surface of burrows and therefore enhances the contrast between burrows and their surroundings. The difference in accuracy between units bgd and Bgd is related to the number of takirs, which leads to a lower accuracy. In unit BgD, the vegetation on the dunes is not yet well developed and easily removed by the gerbils, which leads to very well recognizable burrows.

Mean burrow densities vary between landscape units. The differences are probably even higher in reality, because there is very likely an overestimation of burrows in the units with high TC-Greenness values. Highest burrow density is found in the steppe on inactive floodplain unit (bgd). A possible explanation for this could be the suitable structure of the soil. The lower burrow density in the Bgd floodplain unit is likely due to the higher amount of salt flats, where burrows can hardly be constructed because of the crusted soil. Lower burrow densities in units bGd and BGd might be explained by competition with other rodents if the higher TC-Greenness implies a greater variety of food types and hence opportunities for other species. Interestingly, in the dunes, burrow densities increase with higher TC-Greenness. It might be that in the dunes, the limiting factor is food availability.

The varying burrow densities may imply that also plague dynamics are different across different landscape types, although it is not clear yet how burrow density exactly influences plague dynamics. This is not clear yet, because the question remains whether and how great gerbil movements are related to burrow density, i.e. whether great gerbils move “set” distances or will move to the closest neighbouring burrow, which is of importance for the spread and persistence of plague. Also, burrow density is not necessarily linearly related with great gerbil abundance, since also great gerbil abundance varies per burrow and may also differ per landscape unit.

At the scale of the 200 m research squares, burrow density varies more than between the landscape units. This raises the question at what scale burrow density will vary most and whether this can be related to specific landscape-ecological factors. This topic is part of ongoing research.

### 6.5 Using the created burrow maps for monitoring and the study of plague

The new classification method proposed here is a suitable and helpful tool for the monitoring of the main plague host in the Balkhash area, for example by detecting whether burrows are present in a certain area and where there has been an expansion or reduction of the great gerbils’ territory. Also, the burrow density maps could be combined with field data on burrow occupancy and flea burden, which have been shown to be key elements in predicting outbreaks (Reijniers et al., 2012). This would allow better prediction of where and when outbreaks of plague will occur in great gerbils than currently is possible, certainly if more insight has been obtained in spatial variability of occupancy.

Determining the burrow occupancy from space is an ambitious next objective. The present study found that occupied burrows are easier recognized in images than empty ones. This matches intuitive expectation, since empty burrows will usually have more vegetation growing on the surface of the burrows. Future studies using the current burrow maps to map occupancy, should account for this effect.

The burrow maps can also be used to indicate the risk of transmission of plague to humans, since this risk increases when the animals’ territory encroaches towns and villages. Our maps show for example, that the burrows of the great gerbil are located within 2 km of the main town (Bakanas). In 2010, plague was isolated from great gerbils in an area connected to and less than 5 km away from the Bakanas area, which confirms the necessity for monitoring plague in this area.

## 7 Conclusions

This study presents a new method using object-based image analysis and Random Forests to map burrows across varying landscape types. Classification accuracies are high with producer's and user's accuracies ranging from 88% and 100% in the steppe on inactive floodplain to 86% and 58% in the active floodplain with shrub dominated vegetation. The predicted burrow density for the two images also shows our method performs well: the ratio between predicted and observed burrow density for the whole area is 1.16. Our results are unique: as far as we know, a map of populations of hosts has never been made on such a detailed and extensive spatial scale. The burrow maps are valuable for the monitoring of plague, because they provide a complete overview of the spatial distribution of burrows and allow for the detection of burrows close to human settlements. Moreover, the maps can contribute to epidemiological models and landscape-ecological analysis. Epidemiological modelling could provide more information on whether high density areas provide a higher risk, for example for plague persistence or transmission to humans; the burrow maps are then very useful to identify these high density areas. Our classification method is also suitable for mapping burrows of other rodents, such as marmots and prairie dogs, which are both hosts of plague in other foci.

The object-based stratification method using both high- and medium resolution imagery resulted in improved classification accuracies, especially in the more homogeneous areas. Such a stratified method could well be extrapolated to other studies in complex landscapes.

## Figures and Tables

**Table 1:** Landscape units in the Balkhash basin and the associated field data. The names of the units are built up out of three letters, representing a high (capital letter) or low value (lower case letter) of TC-Brightness (B/b), TC-Greenness (G/g) or DEM-SD (D/d). RF denotes Random Forest.

Landscape unit	Location field data	Number of squares available for RF analysis	Number of burrows available for RF analysis	Stratified RF
bgd	East	6	124	RF bgd-East
Bgd	East and West	24	452	RF Bgd-West and RF Bgd East
bGd	East	16	172	RF bGd East
BGd	–	0*	0*	(Only unstratified RF)
BgD	East and West	6	141	RF BgD West and RF BgD East
bgD	–	0*	0*	(Only unstratified RF)
bGD	East	2	15*	(Only unstratified RF)
BGD	–	0*	0*	(Only unstratified RF)

Total	East and West	54	904	

* No research squares, or too few burrows are located in this landscape unit, so the RF built to classify this area is unstratified, i.e. based on all the squares in the respective SPOT scene.

**Table 2:** Landscape units in the research area.

Landscape unit [TC-Brightness (B/b) TC-Greenness (G/g) DEM-SD (D/d)]	Description	Area cover (%) on the SPOT scenes	Field data	Picture
bgd	Steppe on inactive floodplain	3.4	Yes	Fig. 5a
Bgd	Steppe on inactive floodplain with salt flats	47.5	Yes	Fig. 5b
bGd	Active floodplain with shrub dominated vegetation	22.5	Yes	Fig. 5c
BGd	Floodplain with developing vegetation	1.5	No	–
BgD	Dynamic dunes	11.8	Yes	Fig. 5d
bgD	Stabilized dunes, with inactive vegetation	0.0	No	–
bGD	Stabilized dunes, with active vegetation on moist soil	12.7	Yes	Fig. 5e
BGD	Stabilized dunes, with active vegetation	0.6	No	–

**Table 3:** Classification accuracies of the unstratified (US) and stratified (S) classification per landscape unit. Landscape units BGd, bgD and BGD do not have squares for validation and are therefore excluded from this table.

Landscape unit	Producer's accuracy (%)		User's accuracy (%)		Overall accuracy (%)		Kappa coefficient (^*^significant difference between US and S at *α* = 0.05)		Ratio predicted burrow density: observed burrow density	
	US	S	US	S	US	S	US	S	US	S
bgd	88	88	92	100	91	95	0.81	0.89^*^	0.95 (U)	0.99 (U)
Bgd	77	75	81	81	85	87	0.69	0.70^*^	1.06 (O)	0.99 (U)
bGd	86	86	48	58	87	91	0.54	0.64^*^	1.75 (O)	2.04 (O)
BgD	84	93	77	87	86	91	0.72	0.80^*^	0.94 (U)	0.86 U)
bGD	100	–	60	–	86	–	0.66	–	1.58 (O)	–

Total area	82	–	73	–	86	–	0.67	–	1.16 (O)	–

## Appendix A Supplementary data

The following KML file contains the Google map of the most important area as described in this article.

Map 1 Research Squares.
